# Paraoxonase and Arylesterase Activities in Dipper and Non-Dipper Prehypertensive Subjects

**DOI:** 10.1097/MD.0000000000000786

**Published:** 2015-05-01

**Authors:** Murat Yuksel, Abdulkadir Yildiz, Ebru Tekbas, Ercan Gunduz, Aysun Ekinci, Mehmet Zihni Bilik, Necdet Ozaydogdu, Zuhal Atilgan

**Affiliations:** From the Cardiology Department (MY, AY, ET, MZB, NO, ZA); Internal Medicine/Division of Emergency (EG); and Biochemistry Department, Dicle University School of Medicine, Diyarbakir, Turkey (AE).

## Abstract

Paraoxonase-1, a high-density lipoprotein linked enzyme complex, was shown to be decreased in several cardiovascular diseases. We aimed to explore whether serum paraoxonase and arylesterase activities differ in dipper and non-dipper prehypertensive subjects compared to healthy controls.

Sixty prehypertensive subjects and 30 controls were enrolled. All subjects underwent echocardiographic assessment and 24-hour ambulatory blood pressure monitoring (ABPM). According to the blood pressure (BP) course on ABPM, prehypertensive subjects were categorized into two: non-dipper prehypertensive (NDPH) and dipper prehypertensive (DPH) groups. Serum paraoxonase and arylesterase activities were detected spectrophotometrically.

Paraoxonase and arylesterase activities were significantly lower in patients with NDPH compared to both DPH and control groups. Both paraoxonase and arylesterase activities showed significant negative correlations with BP and left ventricular mass index.

We have demonstrated that NDPH subjects have lower paraoxonase and arylesterase activities compared to DPH subjects and normotensives. Further prospective studies are needed to clarify the role of paraoxonase and arylesterase activities in the development of overt hypertension in prehypertensive subjects.

## INTRODUCTION

The term prehypertension, defined in the 7th Report of the Joint National Committee on the Prevention, Detection, Evaluation, and Treatment of High Blood Pressure (JNC 7), is widely used to classify individuals whose systolic blood pressure (SBP) levels are in the range of 120 to 139 mm Hg and diastolic blood pressure (BP) between 80 and 89 mm Hg.^1^ Individuals with prehypertension are at increased risk for progression to hypertension (at twice risk to develop hypertension as those with lower values).^2,3^ Since prehypertension is a precursor of manifest hypertension (HT), which brings an increased long-term risk for cardiovascular (CV) morbidity and mortality^2,4,5^, prehypertensive subjects require health-promoting lifestyle modifications to prevent CV diseases. Ambulatory blood pressure monitoring (ABPM) provides valuable information about diurnal BP patterns. In the vast majority of the population, night-time BP values are 10% to 20% lower than the day-time BP values (dipper), whereas the nocturnal BP decrease is blunted or even absent in a certain group of people (non-dippers).^6^ The non-dipping BP pattern is also reported to be associated with increased rates of CV morbidity and mortality.^7^

Paraoxonase-1 (PON-1) is an enzyme located on high-density lipoprotein (HDL) particles and was demonstrated to protect lipoprotein particles from oxidation.^8,9^ This enzyme consists of paraoxonase, arylesterase and diazoxonase activities.^10^ Human serum PON-1 activity has been reported to be inversely related to the risk of CV diseases and percutaneous coronary intervention related complications such as no-reflow and contrast induced nephropathy.^11–13^ In addition, reduced PON-1 activities were observed in patients with coronary artery disease, HT, familial hypercholesterolemia and diabetes mellitus.^14–18^

Paraoxonase-1 activity was found to be at low levels in patients with non-dipper HT and white coat HT.^15,19^ However, paraoxonase and arylesterase activities have not been investigated in prehypertension yet. The goal of this study was to evaluate whether or not paraoxonase and arylesterase activities are related to the diurnal BP profiles in prehypertensive subjects.

## METHODS

### Study Population

A total of 286 individuals who admitted consecutively to cardiology outpatient clinic of Dicle University Heart Hospital and underwent 24-hour ABPM between September to December’14 were evaluated for this case–control study. One hundred ninety-six of them were excluded due to exclusion criteria and 60 of them were diagnosed as prehypertension and formed the study group. The remaining 30 healthy individuals having a day-time average SBP < 120 mm Hg and DBP < 80 mm Hg formed the control group. According to JNC 7 report, prehypertensive subjects were defined as those with BPs ranging from 120 to 139 mm Hg systolic and/or 80 to 89 mm Hg diastolic at office settings.^1^ None of the subjects had known coronary risk factors and cardiac symptoms. All participants had normal electrocardiographic and echocardiographic findings. Past medical histories of all participants were recorded and thorough physical examination was performed. The individuals with a history of established heart disease (n = 42), smoking (n = 36), diabetes mellitus (defined as fasting serum glucose level above 126 mg/dL, or receiving oral anti-diabetic medication) (n = 18), HT (defined as BP > 140/90 mm Hg or those who were receiving anti-hypertensive medication) (n = 29), inflammatory diseases (n = 5), cancer (n = 2), chronic renal disease (n = 14), cerebrovascular disease (n = 3), thyroid disease (n = 1), liver disease (n = 3), alcohol or drug abuse (n = 8), hypercholesterolemia (n = 26), steroid use (n = 3) and the ones who refused to participate the study (n = 6) were excluded from the study. The study was approved by the local Ethics Committee, and each subject provided written informed consent.

### Measurement of BP and 24-Hour ABPM

Blood pressures of individuals were measured with a mechanical sphygmomanometer manually in the office settings. The average of two or more properly measured BP was recorded after the individuals were seated quietly for at least 5 minutes in a chair with feet on the floor and arm supported at heart level. Caffeine, exercise, and smoking were avoided for at least 30 minutes prior to BP measurement.

Twenty four-hour non-invasive ABPM was performed on a workday with a portable compact digital BP recorder (Phisio Quant, Envitec Wismar GmbH, Germany). Automatic BP measurements were recorded at 20-min intervals for diurnal readings (08.00–22.00 h) and at 30-minute intervals for nocturnal readings (22.00–08.00 h), which yielded more than 60 BP recordings during the 24-hour period. Night-time and day-time periods were assessed based on the information declared by the subjects. The sleep BP was defined as the average value of the BP measurements from the time subjects went to bed until the time they got up. The day-time BP was defined as the average of the BP recorded during the rest of the day. The subjects were divided into 2 groups according to the amount of decrease in BP during night-time: prehypertensive patients with a nocturnal reduction in average day-time systolic and diastolic BP of less than 10% were classified as non-dipper prehypertensives (NDPH group); those with a ≥10% decrease during night-time were classified as dipper prehypertensives (DPH group).^20–22^

### Echocardiographic Assessment

Transthoracic echocardiography was performed to all participants using an echocardiograph equipped with a broadband transducer (Vivid S6, GE Medical Systems, Horten, Norway). Left ventricle end-systolic (LVESd) and end-diastolic diameters (LVEDd), left atrial diameter, interventricular septal thickness (IVS) and ventricular posterior wall thickness (PW) were measured. Left ventricle (LV) ejection fraction (EF) was determined by Teichholz method.^23^ Left ventricular mass (LVM) was calculated according to the Devereux Formula^24^: LVM = (1.04 × [(LVEDd + IVSth + PWth)^3^ − (LVEDd)^3^] − 13.6). Then, LV mass index (LVMI) was derived by dividing LVM by body surface area.

### Blood Sample Collection

Blood samples were obtained to measure the glucose, creatinine, total cholesterol, triglycerides, LDL, and HDL cholesterol levels following an overnight fasting. Samples were withdrawn from an antecubital vein into dry blood tubes and centrifuged at 3000 rpm for 10 minutes. Serum samples were stored at −80°C and then dissolved and analyzed.

### Paraoxonase and Arylesterase Activity Measurements

Paraoxonase activity was determined as described by Eckerson et al^25^ The rate of hydrolysis of paraoxon was measured by monitoring the increase in absorbance at 412 nm at 25°C. Phenylacetate was used as a substrate to measure the arylesterase activity which is calculated as described by Haagen et al^26^ Both paraoxonase and arylesterase activities were expressed as U/L serum.

### Statistical Analysis

Data were analyzed with the SPSS software version 18.0 for Windows (SPSS, Inc., Chicago, IL). The Kolmogorov–Smirnov test was used to verify the normality of distribution of continuous variables. Continuous variables were defined as mean ± standard deviation; categorical variables were given as percentages. According to the distribution pattern, the independent samples *t* test or Mann–Whitney *U* test was used for the continuous variables between two groups and the chi-square test for categorical variables. Comparison between 3 groups was performed by one-way analysis of variance (ANOVA) or Kruskal–Wallis test. Post hoc analyses of the pair wise comparisons were conducted with Tukey's post hoc test in one-way ANOVA. Mann–Whitney *U* test with Bonferroni correction was used to conduct the pair wise comparisons when there is a significant difference between 3 groups in Kruskal–Wallis test. A *p* value <0.017 (0.05/3) was considered as the cut-off point to reject H_0_ hypothesis during the pair wise comparisons. Pearson or Spearman correlation test was used for correlation analysis. With the help of an online program, the needed sample size for the study was calculated as 30 subjects per group in order to achieve the power of test above 80%. Statistical significance was defined as *p* < 0.05.

## RESULTS

Among 90 patients who were clinically evaluated with the ABPM, 32 patients had non-dipper prehypertension, 28 patients had dipper prehypertension and 30 patients were normotensive. Demographic, clinical, and echocardiographic characteristics of the groups were shown in Table 1. There was no significant difference between the groups in terms of age, gender, body mass index (BMI) and serum glucose, creatinine, and lipid parameters. Left atrial diameter and LVMI of NDPH group were higher than those of both DPH and control groups (*p* < 0.001 and *p* = 0.006, respectively).

**TABLE 1:**
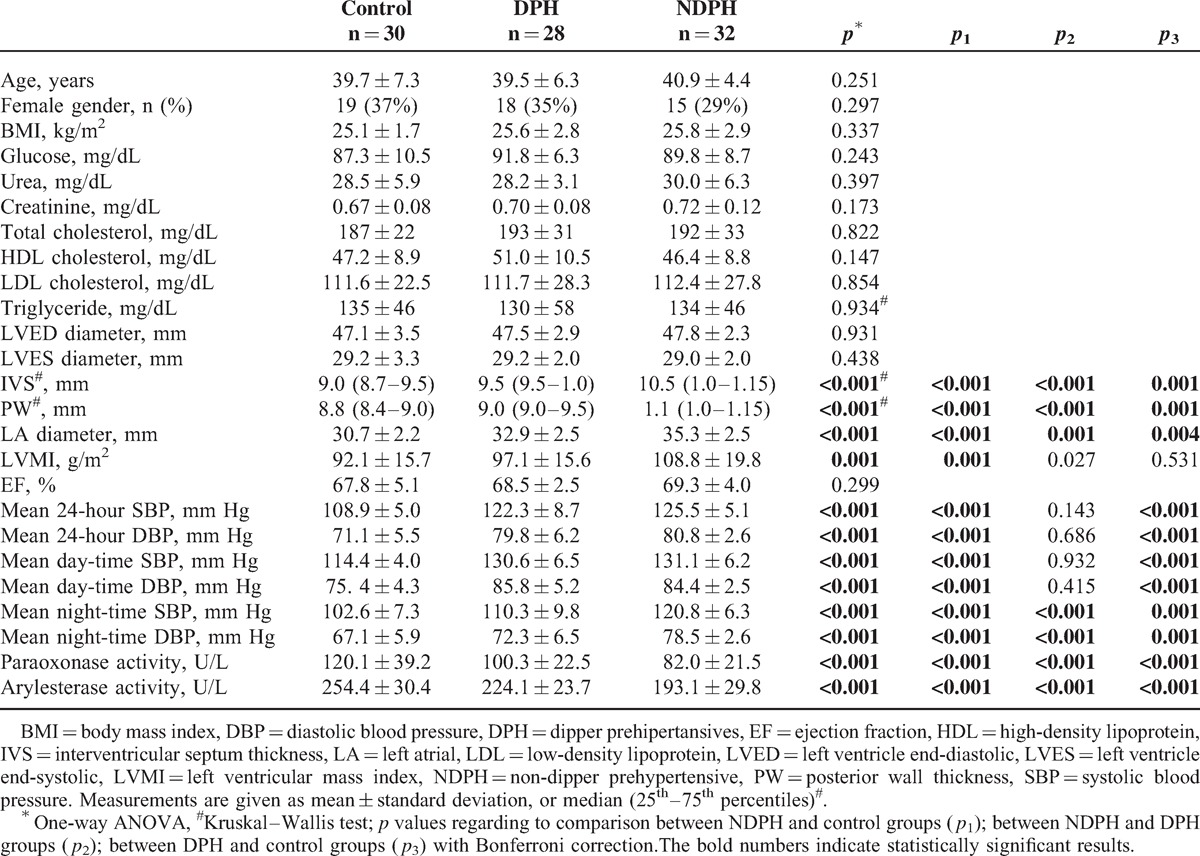
Demographic Data, Laboratory and Echocardiographic Measurements, and Ambulatory Blood Pressure Monitoring Recordings in Study Groups

The measurements of ABPM and paraoxonase and arylesterase activities were shown in Table 1. Both the NDPH and DPH groups have higher mean 24-hour SBP, mean 24-hour DBP, mean SBP daytime, mean DBP daytime, mean SBP night-time, mean DBP night-time than the control group. Twenty-four-hour SBP, 24-hour DBP, mean day-time SBP and mean day-time DBP were not different among DPH and NDPH groups. As expected, mean night-time SBP and mean night-time DBP were significantly higher in the NDPH group than in the DPH group (*p* < 0.001, for both).

**FIGURE 1:**
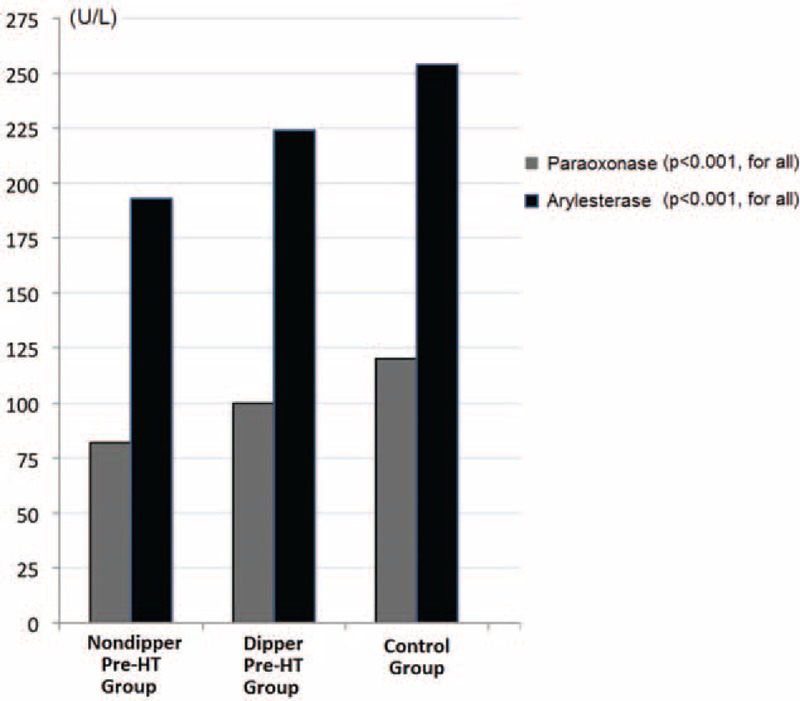
Paraoxonase and arylesterase activities (U/L) of prehypertensive and control subjects.

Serum paraoxonase and arylesterase activities of NDPH group were lower than both DPH and control groups (*p* < 0.001 for all, Figure 1). In addition, DPH group had lower serum paraoxonase and arylesterase activities than those of healthy controls (*p* < 0.001 for all) (Table 1). Paraoxonase and arylesterase activities were positively well correlated with each other (*r* = 0.830, *p* < 0.001). Correlation analysis between enzyme activities and other parameters are presented in Table 2.

**TABLE 2:**
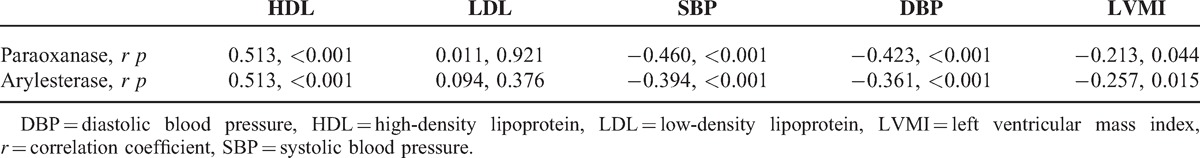
The Results of Correlation Analysis

## DISCUSSION

We evaluated the relation of serum paraoxonase and arylesterase activities with diurnal BP profile in prehypertensive subjects, and showed that mean paraoxonase and arylesterase activities were lower in NDPH group compared to both DPH and control groups. In addition, both paraoxonase and arylesterase activities showed negative correlations with BP and LVMI. Nondipper hypertensives were reported to have greater vascular damage in carotid arteries, higher carotid intima media thickness and more cardiac structural alterations than dipper hypertensives.^20,27,28^ In the present study, NDPH group had significantly higher LVMI than DPH and control groups which was consistent with the previous studies.

Recent studies showed that oxidative stress plays an important role in the atherosclerosis pathogenesis and LDL oxidation has been shown to have a pivotal act in the development of endothelial dysfunction and atherosclerosis.^29,30^ Serum LDL transforms into the oxidized-LDL particle after peroxidation and triggers the endothelial dysfunction, causing platelet aggregation, monocyte adhesion, and increased nitric oxide synthase activity. These events enhance the atherosclerotic process and contribute to the arterial stiffness and HT development.^31,32^ However, HDL can counteract the progression of atherosclerotic process caused by accumulating oxidized-LDL particles. The anti-atherogenic effect of HDL in reverse cholesterol transport is maintained by PON-1.^8^ As PON-1 is an antioxidant enzyme complex located on HDL; it protects both LDL and HDL against oxidation and lowers the levels of lipid peroxides in atherosclerotic lesions.^8,33,34^ Recently, paraoxonase and arylesterase activities were shown to reduce significantly in patients with CAD compared to healthy controls.^35^ Also, Bounafaa et al^36^ showed that paraoxonase, arylesterase and HDL-corrected PON1 activities (PON1 activity/HDL ratio) are significantly lower in patients presented with acute coronary syndrome and paraoxonase and arylesterase activities had a significant protective effect even after adjustment for HDL level, age, BMI, and PON1 polymorphism in the logistic regression analyses. Another study by Demirbag et al^37^ demonstrated reduced levels of paraoxonase and arylesterase activities in CAD patients who received bare-metal stent and developed in-stent restenosis (IRS) compared to those who did not develop IRS and healthy controls. Besides CAD, both paraoxonase and arylesterase activities were found significantly lower in patients with non-dipper HT than those of dipper hypertensives and healthy subjects.^19^ Our results were consistent with previous studies; the NDPH group had lower paraoxonase and arylesterase levels compared to DPH group and healthy subjects.

Several studies have shown that humans with essential HT have a decreased antioxidant capacity and enhanced oxidative stress.^38,39^ Although the exact underlying mechanism of the enhanced oxidative stress in HT is not completely understood it has been widely accepted that increased vascular reactive oxygen species (ROS) forming enzymes, such as xanthine oxidase and NADH/NADPH oxidase, contributes to HT development.^40,41^ Increased ROS in the arterial wall induce smooth muscle contraction and proliferation, which causes aggravation of HT.^42^ Arterial stiffness, evaluated by pulse wave velocity, is an important component of isolated systolic HT in elderly and was shown to be a predictor for future cardiovascular events in several studies.^43,44^ Reduced paraoxonase activity was found to be associated with arterial stiffness in hypertensive and renal transplant patients.^45,46^

The presence of oxidative stress in prehypertensive subjects was reported in previous studies.^47,48^ In a study conducted by Sathiyapriya et al, prehypertensives had higher malondialdehyde and protein carbonyl levels, which are products of polyunsaturated fatty acid peroxidation and protein oxidation, respectively. In addition, erythrocyte glutathione was reduced in the prehypertensive subjects suggesting an imbalance in the oxidant/antioxidant ratio in prehypertensive state.^47^ The ATTICA study that was conducted with 3042 subjects and demonstrated lower total antioxidant capacity and higher oxidized LDL levels in prehypertensive subjects compared to normotensives. Besides, they reported an inverse correlation of total antioxidant status and a positive correlation of oxidized-LDL with BP.^48^ However, previous studies have not investigated paraoxonase and arylesterase activities in prehypertensive subjects. In the present study, paraoxonase and arylesterase activities were decreased in NDPH group compared with DPH and control groups. Moreover, both paraoxonase and arylesterase activities were negatively correlated with BP levels and LVMI.

### Study Limitations

The small sample size and the cross-sectional design were the main limitations of our study. Lack of other oxidative stress markers and PON-1 genotype evaluation was another limitation. However, serum PON-1 activity was shown to be better than PON-1 genotype for the prediction of future CV disease risk.^10^ Our results do not have capability of generalization due to rather small sample size. Further large scale, prospective studies are needed.

## CONCLUSION

In conclusion, with the present study we demonstrated that NDPH subjects have reduced paraoxonase and arylesterase activities compared to DPH subjects and healthy controls. Additionally, NDPH group had significantly higher LVMI compared to DPH and control groups, which may herald forthcoming cardiac structural alterations seen in hypertensive patients. An increase in the oxidant/antioxidant ratio may give an idea about the relation of non-dipping state with increased risk of HT development in the future. With prospective large-scale studies, the role of paraoxonase and arylesterase activities may be clarified in the development of HT in prehypertensive subjects.
